# Effect of insulin sensitivity and secretion defects at mid-pregnancy on early postpartum glucose intolerance in women with gestational diabetes mellitus

**DOI:** 10.3389/fendo.2026.1901350

**Published:** 2026-09-07

**Authors:** Kyung-Soo Kim, Arim Choi, Hyunju Park, Soo-Kyung Kim, Yong-Wook Cho

**Affiliations:** Department of Internal Medicine, CHA Bundang Medical Center, CHA University School of Medicine, Seongnam, Republic of Korea

**Keywords:** gestational diabetes, glucose intolerance, insulin resistance, insulin secretion, insulin sensitivity, postpartum, pregnancy

## Abstract

**Objective:**

This study aimed to evaluate how insulin sensitivity and secretion defects during mid-pregnancy influence the development of early postpartum glucose intolerance in women diagnosed with gestational diabetes mellitus (GDM).

**Methods:**

A total of 1,256 pregnant women diagnosed with GDM between October 2005 and March 2023 were enrolled. Participants were categorized into four groups: GDM-normal, GDM-secretion defect only, GDM-sensitivity defect only, and GDM-mixed defect. Postpartum glucose intolerance was defined as fasting plasma glucose ≥ 100 mg/dL or 2-h plasma glucose ≥ 140 mg/dL during a 75-g oral glucose tolerance test at 6–12 weeks after delivery.

**Results:**

Women presenting with a sensitivity defect at mid-pregnancy showed a significantly higher rate of postpartum glucose intolerance than those without sensitivity defect (64.9% vs. 46.6%, P < 0.001). Conversely, the presence of a secretion defect alone did not result in a significant difference. The highest prevalence of postpartum glucose intolerance was observed in patients with GDM-mixed defect (GDM-normal 45.7%, GDM-secretion defect only 48.5%, GDM-sensitivity defect only 62.8%, GDM-mixed defect 91.3%; P for trend < 0.001). The risk of postpartum glucose intolerance was 1.46 times higher (95% CI 1.07-2.00) for the GDM-sensitivity defect only group and 5.43 times higher (95% CI 1.20-24.56) for the GDM-mixed defect group, compared with the GDM-normal group.

**Conclusions:**

Among women with GDM, impaired insulin sensitivity identified at mid-pregnancy is associated with a substantially increased risk of early postpartum glucose intolerance, whereas insulin secretion defects are not significantly related to this risk.

## Introduction

The global prevalence of gestational diabetes mellitus (GDM) is increasing (1–3). Given its broad negative effects on both mothers and offspring, GDM is recognized as a significant emerging issue in global health (4–7). It is linked to a range of adverse perinatal outcomes, including macrosomia, neonatal hypoglycemia, shoulder dystocia, preeclampsia, and a heightened incidence of cesarean delivery (8, 9). Notably, women with a prior history of GDM face a 50-60% lifetime risk of developing diabetes (10, 11), and those affected by GDM are ten times more likely to progress to type 2 diabetes compared to women without GDM (10). Effective screening and prediction of progression to diabetes among women with GDM are essential for preventive strategies and appropriate long-term follow-up.

As pregnancy progresses to the third trimester, insulin sensitivity may progressively decrease to 50% of the normal expected value (12). GDM is characterized by a compensatory failure of insulin secretion in response to reduced insulin sensitivity during pregnancy (13). Findings from the Genetics of Glucose regulation in Gestation and Growth (Gen3G) cohort indicated that women with GDM who primarily exhibited insulin sensitivity defects at 24–30 weeks’ gestation had altered adipokine profiles, delivered larger infants, and faced an increased risk of GDM-related complications, compared to women with GDM who mainly exhibited insulin secretion defects (14). Abnormalities in insulin sensitivity and secretion during pregnancy may exert a continuous effect on glucose intolerance after childbirth. The importance of postpartum screening for glucose intolerance within 12 weeks after delivery has grown, given the association between GDM and an elevated risk for developing type 2 diabetes post-delivery (15). Nevertheless, limited research has assessed how defects in insulin sensitivity and secretion during pregnancy contribute to postpartum glucose intolerance in women with GDM, particularly in the early postpartum period. This study aimed to assess the influence of mid-pregnancy insulin sensitivity and secretion defects on early postpartum glucose intolerance in women with GDM.

## Methods

### Study population and design

This observational study was conducted at CHA Bundang Medical Center (Seongnam, Korea) from October 2005 to March 2023. We enrolled 1,256 pregnant women aged ≥20 years who were newly diagnosed with GDM. Although a one-step 75-g oral glucose tolerance test (OGTT) approach was proposed in 2010, and adopted in the early 2010s by the American Diabetes Association and the Korean Diabetes Association, a two-step approach was employed to diagnose GDM in this study during the entire study period. At 24 to 28 weeks of gestation, participants were first screened using the 1-hour 50-g glucose challenge test. Women whose 1-hour glucose exceeded 140 mg/dL underwent a 3-hour 100-g OGTT. A diagnosis of GDM was established if at least two out of four plasma glucose measurements (fasting, and at 1, 2, and 3 hours after the 100-g OGTT) surpassed the thresholds specified by the Carpenter and Coustan criteria. Patients with pre-existing type 1 or type 2 diabetes prior to pregnancy, as well as those with twin pregnancies, were excluded.

Anthropometric measurements and laboratory assessments were conducted at 24 to 32 weeks of gestation. Baseline clinical and demographic data, including history of obstetric or medical conditions, family history of diabetes, and pre-gestational body mass index (BMI), were collected from medical records. All participants received medical nutrition therapy during antenatal follow-up. At 6 to 12 weeks postpartum, a 75-g OGTT was administered to determine glycemic status after delivery. The study protocol was approved by the Institutional Review Board of CHA Bundang Medical Center (2017–08–004–001), and written informed consent was obtained from all participants.

### Measurements

Height and weight were assessed with participants wearing minimal clothing and no shoes. Blood pressure was obtained by trained nurses using an automatic sphygmomanometer with participants seated for 10 minutes. All blood samples were drawn following an overnight fast of at least 8 hours, and measurements included fasting plasma glucose, HbA1c, total cholesterol, triglyceride, high-density lipoprotein cholesterol, and insulin. Plasma glucose level was measured using the glucose oxidase method (Hitachi 747 analyser, before January 2007; and Hitachi 7600 analyser, January 2007-March 2016) and enzymatic (hexokinase) method (Cobas 6000 analyzer, after April 2016). Insulin was quantified by radioimmunoassay (COBRA-5010, before February 2006), chemiluminescence immunoassay [DPC 2000 (Immulite), March 2006-December 2008], and electrochemiluminescence immunoassay [Cobas 6000 (e601), January 2009-May 2014; and Cobas 800 (e602) after June 2014]. The homeostasis model assessment of insulin resistance (HOMA-IR) and β-cell function (HOMA-β) were determined according to following formulas (16): HOMA-IR = [fasting insulin (µU/ml) × fasting plasma glucose (mg/dl)]/405; HOMA-β = 20 × fasting insulin (µU/ml)/[fasting plasma glucose (mg/dl) – 63].

### Definition

Insulin secretion and sensitivity were determined by HOMA-β and HOMA-IR, using insulin and glucose values from 24–32 gestational weeks. A HOMA-β in the bottom 25th percentile typically indicates diminished or impaired pancreatic β-cell function, and the 75th percentile for HOMA-IR is often used to establish the baseline for high insulin resistance (17, 18). In this study, an insulin secretion defect was classified when HOMA-β was below the 25th percentile of the cohort, whereas an insulin sensitivity defect was assigned if HOMA-IR was above the 75th percentile. Participants were divided into four subgroups: GDM without either defect (GDM-normal), GDM with only an insulin secretion defect (GDM-secretion defect only), GDM with only an insulin sensitivity defect (GDM-sensitivity defect only), and GDM with both defects (GDM-mixed defect). Large-for-gestational-age was defined as a birth weight above the 90th percentile for gestational age, based on intrauterine growth percentiles derived from the Korean 1999 birth cohort (19). Postpartum glucose intolerance was defined as fasting plasma glucose ≥100 mg/dL or 2-hour plasma glucose ≥140 mg/dL during a 75-g OGTT at 6 to 12 weeks postpartum.

### Statistical analysis

Categorical variables are shown as number (%) and continuous variables as mean ± standard deviation. Comparisons between the groups were tested using the chi-Square test or one-way ANOVA followed by Tukey’s b *post-hot* test, as appropriate. The 25th percentile for HOMA-β was 62.18 and the 75th percentile for HOMA-IR was 1.96 in this population. Pearson’s correlation analysis was used to examine the association between mid-pregnancy HOMA indices and glycemic measures at 6 to 12 weeks postpartum, adjusted for age and pre-pregnancy BMI. Logistic regression was used to calculate odds ratios (ORs) and 95% confidence intervals (CIs) for postpartum glucose intolerance with adjustment for relevant variables. Multivariable models included age, pre-pregnancy BMI, previous GDM, family history of diabetes, parity, and treatment modality for GDM as covariates. A *P*-value less than 0.05 was considered to indicate statistical significance. Analyses were conducted using SPSS version 26.0 (IBM Co., Armonk, NY, USA).

## Results

Baseline characteristics of the study population by insulin sensitivity and secretion defect status at mid-pregnancy are shown in **Table 1**. The mean age was 33.4 years, and the mean pre-pregnancy BMI was 22.5 kg/m^2^. Of the 1,256 women with GDM, 51.9% belonged to the GDM-normal group, 23.2% to the GDM-secretion defect only group, 23.1% to the GDM-sensitivity defect only group, and 1.8% to the GDM-mixed defect group. In the GDM-secretion defect only group, age, pre-pregnancy BMI, blood pressure (systolic and diastolic), HbA1c, lipid profiles, OGTT results (50-g and 100-g), and HOMA-IR were not different from the GDM-normal group. Notably, fasting plasma glucose was higher, while fasting insulin level and HOMA-β were lower in the GDM-secretion defect only group compared to the GDM-normal group. When compared to the GDM-normal group, the GDM-sensitivity defect only group exhibited higher pre-pregnancy BMI, blood pressure (systolic and diastolic), fasting plasma glucose, HbA1c, fasting insulin level, HOMA-IR, and HOMA-β values. Pre-pregnancy BMI, blood pressure (systolic and diastolic), fasting plasma glucose, HbA1c, triglyceride, fasting insulin level, HOMA-IR, and HOMA-β values in the GDM-sensitivity defect only group were higher and HDL-cholesterol was lower than those in GDM-secretion defect only group. Participants in the GDM-mixed defect group were older and demonstrated higher fasting plasma glucose, HbA1c, fasting insulin level, and OGTT (50-g and 100-g) results than the other three groups. Pre-pregnancy BMI and blood pressure (systolic and diastolic) in the GDM-mixed defect group were higher than those in the GDM-normal and GDM-secretion defect group, but were not different from the GDM-sensitivity defect only group.

**Table 1:** Baseline characteristics based on insulin sensitivity and secretion defect status during mid-pregnancy.

Characteristic	Total	GDM-normal	GDM-secretion defect only	GDM-sensitivity defect only	GDM-mixed defect	P value
Number	1,256 (100.0)	652 (51.9)	291 (23.2)	290 (23.1)	23 (1.8)	
Age, yr	33.4 ± 3.7	33.3 ± 3.6	33.2 ± 3.6	33.4 ± 4.1	34.9 ± 4.8^a,b,c^	0.234
Pre-pregnancy BMI, kg/m²	22.5 ± 3.6	21.9 ± 3.1	21.2 ± 2.9	24.8 ± 4.2 ^a,b^	25.0 ± 3.7 ^a,b^	<0.001
History of GDM, %	6.2	5.8	5.8	5.5	30.4	< 0.001
Family history of diabetes, %	34.8	32.1	36.8	37.9	45.5	0.176
Parity (% multiparous)	41.0	39.4	49.8	34.8	52.2	0.001
Systolic blood pressure, mm Hg	111.8 ± 13.3	110.9 ± 13.2	107.4 ± 11.6	117.4 ± 13.1 ^a,b^	119.4 ± 13.1 ^a,b^	<0.001
Diastolic blood pressure, mm Hg	68.2 ± 9.6	67.7 ± 9.3	65.0 ± 8.8	72.1 ± 9.5 ^a,b^	75.3 ± 10.1 ^a,b^	<0.001
Fasting plasma glucose, mg/dL	88.9 ± 14.1	83.2 ± 7.3	89.5 ± 11.0 ^a^	97.0 ± 13.3 ^a,b^	141.1 ± 33.0 ^a,b,c^	<0.001
HbA1c, %	5.3 ± 0.4	5.2 ± 0.4	5.2 ± 0.4	5.5 ± 0.4 ^a,b^	5.7 ± 0.5 ^a,b,c^	<0.001
Total cholesterol, mg/dL	244.8 ± 42.0	246.4 ± 40.2	249.0 ± 44.1	237.6 ± 42.8	231.7 ± 44.3	0.003
Triglyceride, mg/dL	209.2 ± 88.8	208.6 ± 89.2	180.5 ± 60.7	239.2 ± 102.0^b^	219.5 ± 83.7^b^	<0.001
HDL-cholesterol, mg/dL	73.6 ± 13.9	74.5 ± 14.0	75.0 ± 14.1	70.7 ± 13.0^b^	65.0 ± 15.2 ^a,b^	<0.001
Fasting insulin, μU/mL	7.11 ± 6.29	5.59 ± 1.89	3.36 ± 1.32 ^a^	14.11 ± 9.64 ^a,b^	9.06 ± 2.28 ^a,b,c^	<0.001
HOMA-IR	1.66 ± 1.97	1.17 ± 0.44	0.76 ± 0.36	3.53 ± 3.35 ^a,b^	3.12 ± 0.95 ^a,b^	<0.001
HOMA-ß	108.3 ± 121.7	111.6 ± 71.0	46.7 ± 11.4 ^a^	167.4 ± 212.5 ^a,b^	47.4 ± 16.0 ^a,c^	<0.001
50-g OGTT, mg/dL	168.8 ± 22.9	165.6 ± 20.0	171.2 ± 22.9	171.4 ± 24.8	200.6 ± 43.4 ^a,b,c^	<0.001
100-g OGTT						
0 min plasma glucose, mg/dL	95.2 ± 12.6	92.8 ± 11.4	96.4 ± 12.0	98.5 ± 13.1^a^	107.8 ± 23.9 ^a,b,c^	<0.001
60 min plasma glucose, mg/dL	194.5 ± 27.2	192.5 ± 26.1	195.3 ± 26.7	196.2 ± 27.9	224.2 ± 43.8 ^a,b,c^	<0.001
120 min plasma glucose, mg/dL	175.9 ± 26.4	174.5 ± 24.9	174.7 ± 24.9	176.8 ± 27.1	221.5 ± 41.4 ^a,b,c^	<0.001
180 min plasma glucose, mg/dL	145.5 ± 27.3	144.5 ± 26.5	143.7 ± 27.4	147.6 ± 27.4	175.2 ± 46.4 ^a,b,c^	<0.001

Values are expressed as % or mean ± standard deviation.

GDM, gestational diabetes mellitus; BMI, body mass index; HbA1c, hemoglobin A1c; HDL-C, high-density lipoprotein cholesterol; HOMA-IR, homeostasis model assessment of insulin resistance; HOMA-ß, homeostasis model assessment of ß-cell function; OGTT, oral glucose tolerance test.

^a^P<0.05 vs. GDM-normal group

^b^P<0.05 vs. GDM-secretion defect only group

^c^P<0.05 vs. GDM-sensitivity defect only group

Pregnancy outcomes, including gestational age at delivery, preterm delivery, neonatal weight, and the incidence of large-for-gestational-age newborns, did not differ significantly according to the presence of insulin secretion or sensitivity defects (**Table 2**). The frequency of insulin or oral antidiabetic agent usage increased progressively from the GDM-normal group to the GDM-secretion defect only, GDM-sensitivity defect only, and GDM-mixed defect groups. Participants in the GDM-mixed defect group demonstrated higher 75-g OGTT (except 30 min plasma glucose level) results, fasting insulin level, and postpartum HOMA-IR value than the other three groups.

**Table 2:** Pregnancy outcomes and postpartum glycemic parameters based on insulin sensitivity and secretion defect status during mid-pregnancy.

Variable	Total	GDM-normal	GDM-secretion defect only	GDM-sensitivity defect only	GDM-mixed defect	P value
Gestational age at delivery, weeks	38.4 ± 1.7	38.4 ± 1.7	38.5 ± 1.6	38.3 ± 1.7	38.1 ± 1.1	0.427
Preterm delivery, %	11.1	11.3	10.7	11.8	4.3	0.736
Treatment modality for GDM						<0.001
Diet and exercise only, %	80.1	92.2	89.1	72.9	63.9	
Insulin or oral antidiabetic agent, %	19.9	7.8	10.9	27.1	36.1	
Neonate weight, g	3121.5 ± 472.8	3088.4 ± 483.2	3157.3 ± 432.8	3159.7 ± 488.6	3117.6 ± 408.1	0.091
Large-for-gestational-age newborn, %	7.5	6.6	7.9	9.4	4.3	0.470
Postpartum glycemic parameters						
75-g OGTT						
0 min plasma glucose, mg/dL	96.1 ± 9.4	94.3 ± 8.6	96.3 ± 9.4	99.2 ± 9.9 ^a^	105.6 ± 9.0 ^a,b,c^	<0.001
30 min plasma glucose, mg/dL	152.9 ± 24.9	149.5 ± 23.7	155.6 ± 25.6	156.4 ± 25.4	166.4 ± 30.6 ^a^	<0.001
60 min plasma glucose, mg/dL	159.3 ± 36.8	156.4 ± 35.5	160.6 ± 38.0	162.4 ± 37.0	191.2 ± 43.2 ^a,b,c^	0.001
90 min plasma glucose, mg/dL	148.5 ± 38.9	146.9 ± 37.8	148.5 ± 40.0	149.8 ± 38.7	182.9 ± 49.2 ^a,b,c^	0.007
120 min plasma glucose, mg/dL	132.7 ± 31.6	131.8 ± 31.8	133.0 ± 35.1	135.0 ± 35.2	156.6 ± 46.9 ^a,b,c^	0.005
HbA1c, %	5.5 ± 0.4	5.5 ± 0.4	5.5 ± 0.4	5.6 ± 0.4	5.7 ± 0.4 ^a,b^	<0.001
Fasting insulin, μU/mL	5.56 ± 8.38	4.60 ± 4.62	3.82 ± 2.62	8.85 ± 14.23 ^a,b^	13.15 ± 19.26 ^a,b,c^	<0.001
HOMA-IR	1.38 ± 2.36	1.11 ± 1.31	0.93 ± 0.69	2.26 ± 4.00 ^a,b^	3.58 ± 5.66 ^a,b,c^	<0.001
HOMA-ß	60.3 ± 93.4	52.7 ± 45.1	49.3 ± 139.6	84.9 ± 107.6	103.6 ± 125.3 ^a,b^	<0.001

Values are expressed as % or mean ± standard deviation.

GDM, gestational diabetes mellitus; OGTT, oral glucose tolerance test; HbA1c, hemoglobin A1c; HOMA-IR, homeostasis model assessment of insulin resistance; HOMA-ß, homeostasis model assessment of ß-cell function

^a^P<0.05 vs. GDM-normal group

^b^P<0.05 vs. GDM-secretion defect only group

^c^P<0.05 vs. GDM-sensitivity defect only group

**Figure 1:**
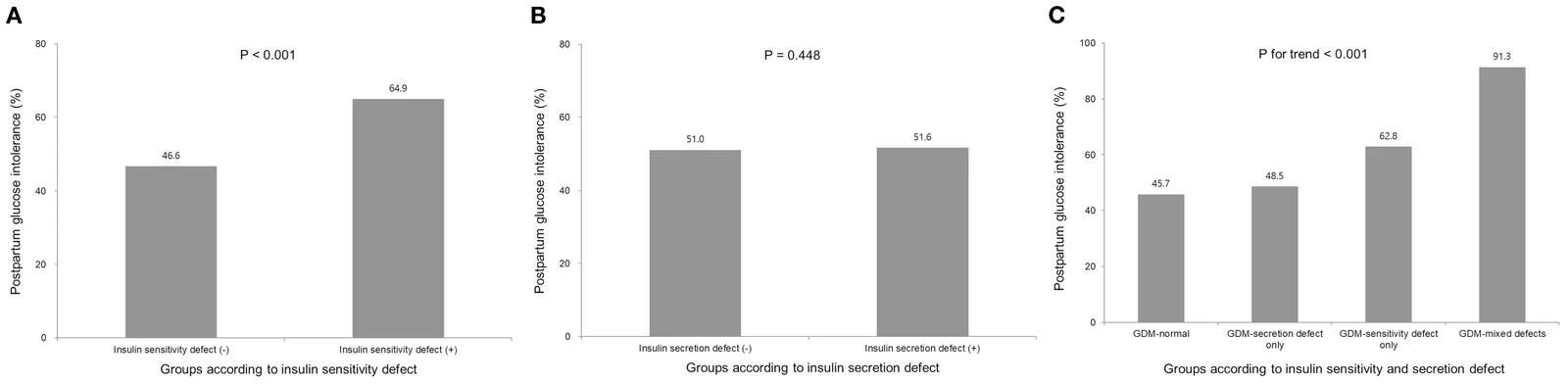
Prevalence of postpartum glucose intolerance stratified by the presence of an insulin sensitivity defect **(A)**, an insulin secretion defect **(B)**, and combined insulin sensitivity and secretion defects **(C)**.

The overall prevalence of postpartum glucose intolerance was 51.1%. Women with a sensitivity defect at mid-pregnancy had a higher rate of postpartum glucose intolerance compared to those without a sensitivity defect (64.9% vs. 46.6%, P < 0.001) (**Figure 1A**). In contrast, there was no significant difference in postpartum glucose intolerance between women with and without secretion defect at mid-pregnancy (**Figure 1B**). The GDM-mixed defect group showed the highest prevalence of postpartum glucose intolerance (GDM-normal 45.7%, GDM-secretion defect only 48.5%, GDM-sensitivity defect only 62.8%, GDM-mixed defect 91.3%; P for trend < 0.001) (**Figure 1C**). At 6 to 12 weeks postpartum, plasma glucose concentrations during the 75-g OGTT and HbA1c also increased sequentially from the GDM-normal to the GDM-secretion defect only, GDM-sensitivity defect only, and GDM-mixed defect groups (**Table 2**). During the early postpartum period, HOMA-IR and HOMA-β values in the GDM-normal group were comparable to those of the GDM-secretion defect only group, while the values in the GDM-sensitivity defect only group were similar to those in the GDM-mixed defect group.

HOMA-IR measured at mid-pregnancy tended to show a positive correlation with 75-g OGTT results performed at 6 to 12 weeks postpartum, with only the 0 min plasma glucose demonstrating statistical significance (**Supplementary Table 1**). Similarly, HOMA-β was significantly correlated only with 0 min plasma glucose, although it generally showed a tendency toward a negative correlation with 75-g OGTT results. HOMA-IR at mid-pregnancy was also positively correlated with postpartum HbA1c after adjustment for age and pre-pregnancy BMI, whereas HOMA-β did not show such a relationship.

In comparison to the GDM-normal group, the GDM-secretion defect only group did not exhibit an increased risk of postpartum glucose intolerance (**Table 3**). The GDM-sensitivity defect only group demonstrated a twofold higher risk of postpartum glucose intolerance when compared to the GDM-normal group (Model 1). Following multivariable adjustment, the GDM-sensitivity defect only group remained at a 1.46-fold (95% CI, 1.07-2.00) increased risk for postpartum glucose intolerance relative to the GDM-normal group (Model 3). Moreover, the GDM-mixed defect group was 5.43 times (95% CI 1.20-24.56) more likely to experience postpartum glucose intolerance compared to the GDM-normal group (Model 3).

**Table 3:** Odds ratios for postpartum glucose intolerance based on mid-pregnancy insulin sensitivity and secretion defect status.

Group	Model 1 (95% CI)	*P* value	Model 2 (95% CI)	*P* value	Model 3 (95% CI)	*P* value
GDM-normal	1.00		1.00		1.00	
GDM-secretion defect only	1.12 (0.85 - 1.47)	0.435	1.23 (0.92 - 1.63)	0.160	1.20 (0.90 - 1.61)	0.209
GDM-sensitivity defect only	2.00 (1.51 - 2.66)	<0.001	1.63 (1.20 - 2.21)	0.002	1.46 (1.07 - 2.00)	0.018
GDM-mixed defect	12.47 (2.90 - 53.63)	0.001	8.97 (2.05 - 39.23)	0.004	5.43 (1.20 - 24.56)	0.028

Model 1: Unadjusted, Model 2: adjusted by age and pre-pregnancy body mass index, Model 3: adjusted by age, pre-pregnancy body mass index, history of gestational diabetes mellitus, family history of diabetes, parity, and treatment modality for gestational diabetes mellitus.

CI, confidence interval; GDM, gestational diabetes mellitus.

## Discussion

This study demonstrated that impaired insulin sensitivity at mid-pregnancy was associated with an elevated risk of early postpartum glucose intolerance in women with GDM, whereas an insulin secretion defect was not. Women presenting with a sensitivity defect at mid-pregnancy exhibited a higher prevalence of postpartum glucose intolerance compared to those without such a defect, while no significant difference was observed between women with and without a secretion defect. The GDM-sensitivity defect only and GDM-mixed defect groups had a higher risk of postpartum glucose intolerance compared to the GDM-normal group, whereas there was no increased risk observed for the GDM-secretion defect only group. Notably, this is the first study to specifically assess the impact of insulin sensitivity and secretion defect at mid-pregnancy on early postpartum (6 to 12 weeks after delivery) glucose intolerance in women with GDM.

Multiple factors contribute to the increased risk of postpartum glucose intolerance in women with GDM (20–22). A meta-analysis of 37 studies identified several risk factors, such as family history of diabetes, insulin treatment during pregnancy, fasting and 1-h glucose levels at 24–28 weeks of gestation, pre-pregnancy BMI, triglyceride levels during 28–40 weeks of gestation, HbA1c levels at 28–40 weeks of gestation, and a history of previous GDM as being associated with a higher risk for glucose intolerance at 6–12 weeks postpartum in patients with GDM (20). Our previous research indicated that vitamin D deficiency at mid-pregnancy was linked to an increased risk of postpartum glucose intolerance in women with GDM (22). Since insulin secretion and sensitivity change dynamically throughout pregnancy, these alterations can influence the development of postpartum glucose intolerance (12). However, to date, the effects of insulin sensitivity and secretion defect at mid-pregnancy on early postpartum glucose intolerance in women with GDM have not been thoroughly investigated.

Generally, defects in either insulin secretion or insulin sensitivity are recognized as primary factors driving the progression to type 2 diabetes mellitus (23, 24). In comparison to Western populations, multiple studies have highlighted β-cell dysfunction as a key element in the pathogenesis of diabetes among Asian individuals (25, 26). A prospective cohort study of 4,106 Korean participants reported that impairment in β-cell function was a stronger predictor of incident diabetes over a 10-year follow-up period than decreased insulin sensitivity (26). Moreover, trajectory analysis indicated that the decline in insulin sensitivity among subjects progressing to diabetes was not adequately compensated by increased β-cell function (26). By analogy, for women with GDM, it would be anticipated that impaired insulin secretion confers a higher risk of postpartum glucose intolerance. Unexpectedly, our study demonstrated that impaired insulin sensitivity rather than an insulin secretion defect during mid-pregnancy is more strongly associated with an increased risk of early postpartum glucose intolerance in women with GDM. To date, there have been no investigations specifically assessing the roles of insulin sensitivity and secretion defects during pregnancy in predicting postpartum glucose intolerance among GDM patients. In a study involving 809 women at 24–30 weeks’ gestation, including 67 with GDM, nearly one-third of GDM cases exhibited predominant insulin secretion defects without evidence of impaired insulin sensitivity, while roughly half displayed predominant insulin sensitivity defects with hyperinsulinemia (14). In that study, women with GDM characterized by predominant insulin sensitivity defects—as opposed to those with predominant secretion defects—exhibited altered adipokine profiles, delivered larger infants, and faced a greater risk of GDM-related complications (14). The underlying explanation for these differences observed between general and pregnant populations has not been fully elucidated. It is possible that, as insulin secretion defects during pregnancy might be relative rather than absolute, defects in insulin sensitivity emerge as a more significant determinant of postpartum glucose intolerance. Additional prospective studies are needed to clarify the impact of insulin sensitivity and secretion defects at mid-pregnancy on early postpartum glucose intolerance in women with GDM.

Pregnancy is characterized by numerous metabolic, biochemical, and physiological alterations. Pregnant women require an additional 300 kcal/day beyond the typical energy intake to accommodate glucose demands for the developing fetus (27). As a result, the emergence of decreased insulin sensitivity, known as insulin resistance, is a physiological adaptation that ensures sufficient carbohydrate availability for the rapid fetal growth (28). The reduction in insulin sensitivity during pregnancy has been attributed to multiple factors, including estrogen, progesterone, and human placental lactogen, among others (29). It is well-established that women who develop GDM exhibit abnormally increased insulin resistance resulting from alterations in insulin signaling pathways, disruption of normal subcellular localization of GLUT4 transporters, elevated expression of the membrane glycoprotein PC-1, or diminished insulin-mediated glucose transport (30). The presence of impaired insulin sensitivity during pregnancy is additionally linked to complications such as premature labor, antepartum or postpartum hemorrhage, and fetal issues such as intrauterine growth retardation, fetal overgrowth, and prematurity (31). Moreover, it elevates the long-term risk of developing metabolic syndrome, diabetes mellitus, hypertension, hyperlipidemia, and cardiovascular diseases (32). Consistent with previous investigations, our study demonstrated that impaired insulin sensitivity at mid-pregnancy was associated with an increased risk of early postpartum glucose intolerance in women with GDM.

Although it is not yet clearly known, the possible mechanism how insulin sensitivity defect cause postpartum glucose intolerance were adipose tissue dysfunction, inflammation, lipotoxicity, and so on (33, 34). Adipokines including adiponectin, leptin, adipocyte fatty acid-binding protein, resistin, visfatin, omentin, vaspin, apelin, and chemerin secreted by adipose tissue, may contribute directly and/or indirectly aggravating insulin resistance and promoting postpartum glucose intolerance onset, through the enhancement of chronic inflammation (33). Low grade inflammation can interfere with insulin signaling pathways, leading to defective GLUT4 translocation and reduced glucose uptake in peripheral tissues (34). Further studies are warranted to figure out the mechanism between insulin sensitivity defect and postpartum glucose intolerance.

Several limitations should be acknowledged when interpreting the findings of this study. First, we did not assess insulin sensitivity and secretion abnormalities with gold standard methodologies. Nonetheless, HOMA-IR and HOMA-β are well-validated and widely utilized glycemic indices, which support their use in numerous epidemiological investigations. Second, due to the absence of data beyond 6 to 12 weeks postpartum, we were unable to examine the influence of mid-pregnancy insulin sensitivity and secretion impairment on later postpartum glucose status. Third, as our cohort consisted exclusively of Korean women with singleton pregnancies, extrapolation of these results to other ethnic groups remains limited. Fourth, currently, both one-step and two-step approaches are used for the diagnosis of GDM, but in this study, only the two-step approach was used for consistency. Fifth, because the number of the GDM-mixed defect group was small, an OR of the GDM-mixed defect group had a wide CI. Lastly, the study sample was relatively small and not entirely representative of the Korean population, underscoring the need for larger prospective studies to verify these observations. Despite these limitations, this study provides valuable insights by evaluating the impact of mid-pregnancy insulin sensitivity and secretion defects on early postpartum (6 to 12 weeks following delivery) glucose intolerance among women with GDM.

In summary, decreased insulin sensitivity, rather than impaired insulin secretion, at mid-pregnancy was linked to a heightened risk of early postpartum glucose intolerance in women with GDM. These data suggest the importance of assessment for insulin secretion and sensitivity in women with GDM. Therefore, it is important to assess postpartum glucose intolerance in women with GDM who exhibit reduced insulin sensitivity during mid-pregnancy.

## Data Availability

The original contributions presented in the study are included in the article/**Supplementary Material**. Further inquiries can be directed to the corresponding author.
