# Supplementary material for: Effect of insulin sensitivity and secretion defects at mid-pregnancy on early postpartum glucose intolerance in women with gestational diabetes mellitus
Source: Front Endocrinol (Lausanne). 2026 Sep 7;17:1901350. doi: 10.3389/fendo.2026.1901350 (PMC13590348; doi:10.3389/fendo.2026.1901350)
Supplement: Supplementary file 1 [file Table1.docx]

**Supplementary Table 1** Correlation between HOMA indices during mid-pregnancy and glycemic parameters at 6 to 12 weeks postpartum

|  | HOMA-IR | | | | HOMA-ß | | | |
| --- | --- | --- | --- | --- | --- | --- | --- | --- |
| Variable | Unadjusted | | Adjusted | | Unadjusted | | Adjusted | |
|  | *r* | *P* value | *r* | *P* value | *r* | *P* value | *r* | *P* value |
| 75-g OGTT |  |  |  |  |  |  |  |  |
| 0 min plasma glucose, mg/dL | 0.170 | <0.001 | 0.101 | 0.003 | -0.082 | 0.004 | -0.111 | 0.001 |
| 30 min plasma glucose, mg/dL | 0.067 | 0.039 | 0.056 | 0.100 | -0.022 | 0.498 | -0.027 | 0.424 |
| 60 min plasma glucose, mg/dL | 0.078 | 0.015 | 0.053 | 0.119 | 0.007 | 0.825 | -0.001 | 0.984 |
| 90 min plasma glucose, mg/dL | 0.051 | 0.121 | 0.013 | 0.698 | 0.007 | 0.841 | -0.005 | 0.894 |
| 120 min plasma glucose, mg/dL | 0.045 | 0.110 | 0.011 | 0.737 | -0.012 | 0.682 | -0.021 | 0.531 |
| HbA1c, % | 0.102 | <0.001 | 0.067 | 0.020 | -0.011 | 0.693 | -0.024 | 0.410 |
| HOMA-IR | 0.169 | <0.001 | 0.102 | 0.003 | 0.085 | 0.003 | 0.076 | 0.026 |
| HOMA-ß | 0.128 | <0.001 | 0.074 | 0.031 | 0.147 | <0.001 | 0.140 | <0.001 |

adjusted by age and pre-pregnancy body mass index.

HOMA-IR, homeostasis model assessment of insulin resistance; HOMA-ß, homeostasis model assessment of ß-cell function; HbA1c, hemoglobin A1c; OGTT, oral glucose tolerance test.
